# NP方案治疗1例肺低分化癌伴肉瘤样改变病例报道

**DOI:** 10.3779/j.issn.1009-3419.2011.06.13

**Published:** 2011-06-20

**Authors:** 洋 焦, 冲 白

**Affiliations:** 200433 上海，第二军医大学附属长海医院呼吸内科 Department of Respiratory, Changhai Hospital, Second Military Medical University, Shanghai 200433, China

肺肉瘤样癌（pulmonary sarcomatoid carcinoma, PSC）是一种少见的肺部肿瘤，恶性程度高，目前国内外对PSC化疗方案的选择及疗效的报道较少。本文报道1例有远处转移的肺低分化癌伴肉瘤样改变的病例经过2次NP方案（长春瑞滨+顺铂）治疗后取得部分缓解。

## 1 病例资料

患者，男性，52岁，因“咳嗽咳痰4月余”于2009年5月24日入第二军医大学附属长海医院。患者于2009年1月初无明显诱因出现阵发性干咳，后逐渐加重，伴活动后气喘。当地医院以抗感染、平喘等对症治疗效果欠佳。2009年5月初外院胸片示右下肺肿块，为进一步治疗前来第二军医大学附属长海医院。既往无特殊病史，吸烟指数400年支。入院后查体：全身浅表淋巴结未扪及肿大，两肺呼吸音稍低，未闻及干湿啰音。心腹部查体无明显异常。2009年5月25日胸部增强CT（图 1A）示右肺下叶后段见一7 cm×7 cm大小肿块，分叶及毛刺，右中间支气管内见软组织影，下肺及右中肺见小片状模糊影，右侧肺门及气管隆突下见淋巴结肿大。头颅MRI增强及腹部彩超未见明显异常。骨ECT示T12、L4、右髂臼上缘、左骶髂关节下部转移灶。腰椎MRI示胸12、腰5椎体及腰4棘突见不规则骨质破坏，可见T1WI、T2WI低信号，增强后局部低信号，腰4棘突处软组织内长T2信号，增强后不均匀强化。血CEA为320.7 ng/mL，NSE为21.37 μg/L。2009年5月25日气管镜检查见右中间支气管新生物，于此处高频圈套（40 w）取得组织后送检。病理检查见梭形、短梭形肿瘤细胞片状排列，核异形，部分多角形细胞，巢状排列，肿瘤间质粘液变形（图 2A）；免疫组化（图 2B，图 2C）：CK8/18（上皮+）、肌动蛋白结合蛋白CALP（-）、EMA（上皮+-）、CK7（-）、VIM（梭形细胞+）、P63（+）、Des（梭形细胞+）、TTFI（+）、平滑肌肌动蛋白SMA（-）、Chr（-），诊断为右肺中间支气管开口低分化上皮性恶性肿瘤伴肉瘤样癌改变。临床诊断为右下肺低分化上皮性恶性肿瘤伴肉瘤样改变T4N2M1（骨）Ⅳ期。

**1:**
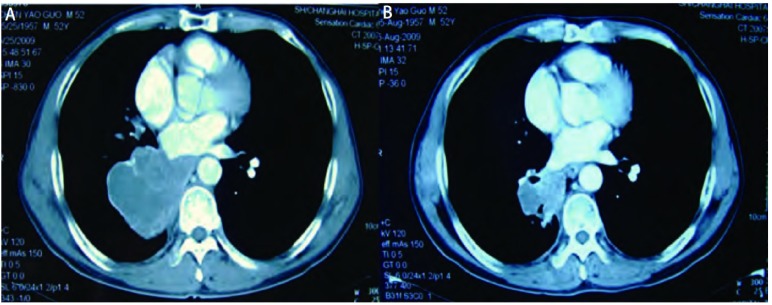
患者治疗前后胸部影像学改变。A：治疗前右下肺肿瘤最大直径为7 cm；B：两次化疗后肿瘤最大直径为3 cm。 The appearance of the chest computed tomogram. A: The diameter of tumor in right low lung was 7 cm before chemotherapy; B: The diameter of tumor in right low lung was 3 cm after two times chemotherapy.

**2:**
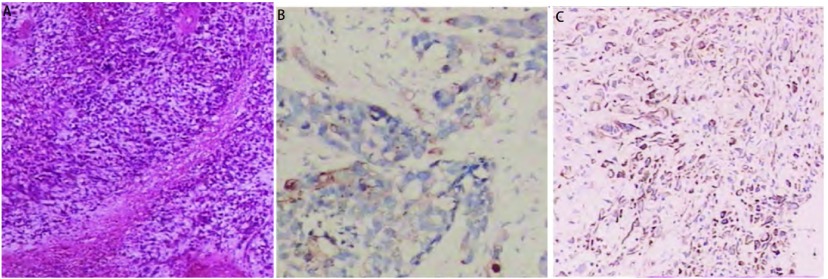
病理表现。A：肿瘤细胞形态多样，以小圆形、短梭形为主，形态较幼稚，弥漫杂乱排列，可见较多核分裂像及坏死组织（HE, ×100）；B：CK8表达阳性（Envision，×200）；C：Vimentin表达阳性（Envision，×100）。 The appearance of pathological findings. A: the morphous of tumor cell is variety, the main form is round and spindle with immature, which can saw karyokinesis and necrosis tissues (HE, ×100); B: CK8 positive expression (Envision, ×200); C: Vimentin positive expression (Envision, ×100).

2009年5月30日予NP方案（长春瑞滨40 mg d1，d5+顺铂40 mg d1-d3）化疗，21天为1周期，按疗程化疗2次后于2009年8月5日复查。血CEA为154 ng/mL，胸部增强CT（图 1B）见右肺下叶病灶较前明显缩小，对比入院时胸部CT结果，其肺内原发灶最大单径由7 cm缩小到3 cm，缩小率>30%，根据RECIST标准病情评估为部分缓解，随即完成第3次化疗。但患者化疗后骨髓抑制较严重，血白细胞总数波动在（2.5-3.5）×10^9^/L，血红蛋白80 g/L-110 g/L，渐出现腰痛症状。2009年9月11日及10月19日分别完成了第4次、第5次化疗，并每隔4周予唑来膦酸注射液4 mg（天晴依泰）静滴抑制骨破坏。2010年2月复查胸部CT，肺内病灶大小较前无明显改变。但患者因身体耐受及经济情况未再行进一步治疗。出院后电话随访获知约3个月后去世。

## 2 讨论

PSC是一种少见的肺部原发肿瘤，占肺部非小细胞癌肿瘤的0.1%-0.4%。2004年WHO肺肿瘤组织学分类^[1]^将PSC定义为具有多形性、肉瘤样或肉瘤成分的癌，包括具有梭形和（或）巨型细胞的癌、癌肉瘤、肺母细胞瘤。对于肉瘤样癌的起源，过去认为是分别由癌细胞和肉瘤样细胞分化组织形成，也有认为由原始的全能干细胞分别向癌组织和肉瘤样组织分化形成。近年来随着免疫组化技术的应用，发现肉瘤样成分中多表达上皮性标志物，同时癌成分中也表达间叶性标志物（如波形蛋白），越来越多的研究表明肉瘤样癌实则仍为上皮性肿瘤。目前采用Nappi等^[2]^对PSC的诊断标准将其分为单相性肿瘤（由肉瘤样梭形细胞或巨细胞组成）和双相性肿瘤（由恶性上皮成分和恶性间叶成分组成，不包括特殊间叶成分，如骨、软骨、横纹肌肉瘤）。

PSC患者的中位年龄在60岁-65岁，男性居多，与吸烟关系密切。临床症状以咳嗽、咳痰、痰中带血多见，其它包括胸痛、乏力、气短和发热症状。周围型肉瘤样癌的CT增强后多可见不均匀强化。因其恶性程度高，对于放疗和化疗不敏感，预后较其它类型肺癌差。有学者^[3]^总结了美国MD Anderson肿瘤中心20年来资料完整的63例肺肉瘤样癌患者的结果，肺肉瘤样癌和匹配的非小细胞肺癌（non-small cell lung cancer, NSCLC）患者的5年生存率分别为24.5%和46.3%（*P*=0.01），进一步分层分析显示只有Ⅲ期PSC患者的生存期较匹配的NSCLC患者缩短（中位生存期分别为10.3个月和25.3个月，*P*=0.006）。徐文静等^[4]^对47例PSC患者进行预后因素的分析，认为年龄、肿瘤大小、pTNM分期、T1-T2期与T3-T4期、有无淋巴结转移、M分期和组织学类型对生存期的影响有统计学意义，但仅年龄、T分期和M分期是影响预后的独立因素，吸烟与预后关系不大，Ⅰ期-Ⅲ期及是否行术后化疗患者的1年、5年生存率差异无统计学意义，仅3年生存率差异有统计学意义，而伴有远处转移的1年生存率为0。Yuki等^[5]^则认为淋巴结转移状况是最重要的预后影响因素，与N0期的患者相比N1/N2患者的总生存期和无瘤生存期均明显缩短。

目前认为，对于肉瘤样癌患者的首选治疗方法为手术切除。是否行放疗和化疗以及选择何种治疗方案的报道较少，疗效也不确切。国内有报道^[6]^1例术后复发的PSC患者予TP方案（紫杉醇+顺铂）化疗后达到部分缓解，而另1例Ⅳ期的患者经吉西他滨联合顺铂和口服吉非替尼（易瑞沙）治疗后仍有进展。Raveglia等^[7]^报道20例PSC患者中6例Ⅰ期患者未行化疗，14例Ⅱ期、Ⅲ期患者术后接受了铂类为基础的化疗，结果显示中位生存期分别为26个月和7个月，提示由于PSC恶性程度高和预后差的特点，是否应用化疗值得考虑。但作者同时也表明由于病例数少，特别是缺乏Ⅳ期患者的病例资料，尚不能正确评价辅助化疗在PSC治疗中的作用。

表皮生长因子受体（epidermal growth factor receptor, EGFR）是Ⅰ型跨膜受体酪氨酸激酶Erb家族的一员，在很多上皮肿瘤细胞中过度表达，其中NSCLC中的表达率为40%-80%^[8]^。EGFR的生物靶向治疗对于NSCLC特别是腺癌有着越来越重要的价值。但EGFR抑制剂类的药物对于PSC的治疗效果尚不明确。Italiano等^[9]^对22例PSC患者的肿瘤组织进行了EGFR蛋白表达、*EGFR*基因拷贝数、*EGFR*突变及*KRAS*突变的检测，结果显示EGFR蛋白表达率为100%，远远高于文献^[10]^报道的典型NSCLC中由相同的评分系统得出的比例50%-70%，*EGFR*基因拷贝数增长率为23%，KRS突变率为38%，而*EGFR*突变率为0，因此作者认为相对于典型的NSCLC，PSC患者中*KRAS*高突变率、EGFR蛋白的过表达可能与其较差的预后有关，而无*EGFR*突变提示PSC患者可能无法获益于抗-EGFR的靶向治疗。但Leone等^[11]^则认为Italiano等^[9]^报道的8例*KRAS*突变的病例中有2例的检测方法并非经典的直接测序法，存在一定误差，因此*KRAS*基因的突变率应为28.5%（6/22），这与其它NSCLC的报道^[12]^无明显差异，而*EGFR*的基因突变也可以发生在PSC中。Ushiki等^[13]^报道了1例吉非替尼治疗PSC的病例，尽管此例患者没有从靶向治疗中受益，但在尸检中发现其*EGFR*基因中的外显子19缺失，外显子20突变。Leone等^[11]^亦对22例PSC进行了*EGFR*相关检测，其中2例患者存在*EGFR*突变。综合上述作者的结论，在PSC患者中可以存在*EGFR*突变，但几率很小，而*EGFR*突变的患者也并非都能从中受益。EGFR蛋白的过表达及*EGFR*基因的低突变率可能与PSC病情发展迅速以及治疗效果欠佳存在一定关系。

本例患者的发病年龄、吸烟史、临床症状以及肿块大小、形态、免疫组化标记物的成分都符合PSC的一般特点。其特殊性在于该病例是有远处转移的Ⅳ期患者，经过2个疗程的NP方案化疗后病情评估为部分缓解，随后多次复查病情均为稳定。相关文献中Ⅳ期PSC的病例较少，对于此类患者行化疗后取得明显疗效的病例更鲜有报道。推测原因在于PSC无特定的临床表现，多由术后病理明确，故晚期、特别是有转移的、无手术机会的确诊例数较少，缺乏化疗方案治疗的大宗病例报道。因此，对于化疗及生物治疗对肉瘤样癌肿的作用还有待于*meta*分析或更完善的实验研究来进一步明确。

## References

[1] Brambilla E, Travis WD, Colby TV (2001). The new World Health Organization classification of lung tumors. Eur Respir J.

[2] Nappi O, Glasner SD, Swanson PE (1994). Biphasic and monophasic sarcomatoid carcinomas of the lung. A reappraisal of 'carcinosarcomas' and 'spindlecell carcinomas'. Am J Clin Pathol.

[3] Venissac N, Pop D, Lassalle S (2007). Sarcomatoid lung cancer (spindle/giant cells): an aggressive disease?. J Thorac Cardiovasc Surg.

[4] Xu WJ, Huang C, Wang L (2008). Characteristics and prognostic analysis of 47 patients with lung sarcomatoid carcinoma. Chin J Clin Oncol.

[5] Yuki T, Sakuma T, Ohbayashi C (2007). Pleomorphic carcinoma of the lung: a surgical outcome. J Thorac Cardiovasc Surg.

[6] Jiang M, Cao D, Yang Y (2006). Clinical analysis of pulmonary sarcomatoid carcinoma. Chin J Lung Cancer.

[7] Raveglia F, Mezzetti M, Paniqalli T (2004). Personal experience in surgical management of pulmonary pleomorphic carcinoma. Ann Thorac Surg.

[8] Minna J D, Dowell J (2005). Erlotinib hydrochloride. Nat Rev Drug Discov.

[9] Italiano A, Cortot AB, Ilie M (2009). EGFR and KRAS status of primary sarcomatoid carcinomas of the lung: implications for anti-EGFR treatment of a rare lung malignancy. Int J Cancer.

[10] Hirsch FR, Varella-Garcia M, Bunn PA Jr (2003). Epidermal growth factor receptor in non-small-cell lung carcinomas: correlation between gene copy number and protein expression and impact on prognosis. J Clin Oncol.

[11] Leone A, Graziano P, Gasbarra R (2011). Identification of EGFR mutations in lung sarcomatoid carcinoma. Int J Cancer.

[12] Linardou H, Dahabreh IJ, Kanaloupity D (2008). Assessment of somatic *k-Ras* mutations as a mechanism associated with resistance to EGFR targeted agents: a systematic review and *meta*-analysis of studies in Advanced nonsmall cell lung cancer and metastatic colorectal cancer. Lancet Oncol.

[13] Ushiki A, Koizumi T, Kobayashi N (2009). Genetic heterogeneity of *EGFR* mutation in pleomorphic carcinoma of the lung: response to gefitinib and clinical outcome. Jpn J Clin Oncol.

